# Assembly of the Linear Viral Nucleocapsid

**DOI:** 10.3390/microorganisms14040848

**Published:** 2026-04-09

**Authors:** Ming Luo, Kristin V. Lyles, Oluwafoyinsola O. Faniyi, Ryuha Kim

**Affiliations:** 1 Department of Chemistry, Georgia State University, Atlanta, GA 35302, USA; kvanmouwerik1@gsu.edu (K.V.L.); ofaniyi1@gsu.edu (O.O.F.); rkim45@gsu.edu (R.K.); 2 Center for Diagnostics and Therapeutics, Georgia State University, Atlanta, GA 35302, USA

**Keywords:** nucleocapsid, negative strand RNA virus, linear symmetry, capsid protein, RNA encapsidation, viral RNA synthesis

## Abstract

Nucleocapsids protect viral genomes and play fundamental roles in viral assembly and infection. While many viruses adopt icosahedral or helical symmetries, negative-strand RNA viruses (NSVs) assemble their nucleocapsids with a distinct translation-based symmetry that is often considered helical because of their curvature. Our study analyzes the structural basis, assembly principles, and functional implications of the linear nucleocapsids. Structural coordinates of viruses were obtained from the Protein Data Bank (PDB) and examined using PyMOL version 1.3 to compare protein folds, RNA–protein interactions, inter-subunit contacts, and curvature properties across multiple nucleocapsids. We found that linear nucleocapsids share a similar 5H + 3H fold in their capsid proteins and encapsidate a fixed number of nucleotides per subunit, though the degree of nucleotide sequestration varies. Their architecture differs in inter-subunit interactions, determining whether empty capsids can assemble and influencing RNase sensitivity. Although these nucleocapsids may appear helical, they lack strict helical symmetry and instead display variable curvature that is modulated by environmental conditions. Relaxation of this curvature is likely required for viral RNA-dependent RNA polymerase to access the sequestered RNA genome during transcription/replication. In conclusion, linear nucleocapsids constitute a class of RNA–protein assemblies with variable curvature. The topologically conserved fold of the capsid protein enables genome protection while regulating exposure of RNA during viral RNA synthesis.

## 1. Introduction

All viruses contain a protein capsid that packages a nucleic acid genome and protects it during transmission. This fully assembled complex of the protein capsid with the nucleic acid genome is termed the nucleocapsid. The nucleocapsid allows for the construction of an infectious virus particle together with other components required for virus entry, including the viral membrane envelope, receptor-binding proteins, and fusion proteins, to successfully infect the host cell.

The protein capsid is composed of protein subunits arranged according to certain geometrical symmetry. One common symmetry is the icosahedral symmetry that appears to be spherical, with 12 vertices, 20 faces, and 30 edges [1,2]. In some cases, it is an elongated icosahedral capsid. The volume of the icosahedral capsid is fixed by its defined symmetry, allowing only a maximal length of the nucleic acid genome to be packaged. Examples include the head of bacteriophages, herpes virus, and picornaviruses. Another common symmetry is the helical symmetry with a fixed number of protein subunits per turn [1,2]. There is also a fixed number of nucleotides associated with one protein protomer (subunit) that follows the same helical symmetry. The size of the helical nucleocapsid is determined by the length of the nucleic acid genome when the entire genome is covered by the capsid proteins. The cylindrical nucleocapsid of tobacco mosaic virus and M13 phage represents this typical helical symmetry.

**Figure 1:**
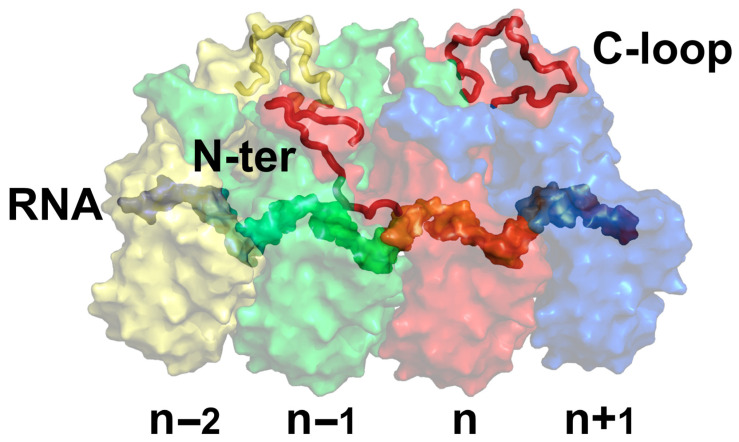
Structure of VSV Nucleocapsid. Four consecutive capsid subunits are shown, with the position of each subunit labeled below. The encapsidated RNA is sequestered within the capsid proteins. The N-terminus and the C-loop of subunit (n) are highlighted (red). The C-loop of subunit (n − 2) is also highlighted (yellow). Subunit (n − 1) is shown in green, and subunit (n + 1) is shown in blue. The C-terminal domains (CTDs) are above the RNA, whereas the N-terminal domains (NTDs) are below the RNA. Coordinates used for the drawing are derived from PDB 7UWS [20].

Another class of nucleocapsids exhibits a less recognized symmetry. These are linear nucleocapsids assembled with translational symmetry. By comparing a large number of reported structures of these nucleocapsids determined by cryoEM/cryoET [3,4,5,6,7,8,9] we can unveil the assembly principles of these linear nucleocapsids. The capsid protein subunits maintain the same orientation and are packed side-by-side along the length of the nucleic acid genome (Figure 1). Each subunit has the same association with a fixed number of nucleotides, repeating the same pattern when translated by one subunit along the length of the genome. This type of linear nucleocapsid is often inaccurately treated as helical because it may exhibit a helical appearance. In a strict helical nucleocapsid, such as that of tobacco mosaic virus, the nucleocapsid is disassembled if the helical symmetry is disrupted [10,11]. In this type of virus, the genomic RNA must be released by uncoating (or disassembling) the helical nucleocapsid prior to viral RNA synthesis [12,13,14,15]. In a linear nucleocapsid, however, the degree of helical curvature may change when the environment surrounding the nucleocapsid is changed, such as a change in pH or ionic strength, without disassembly of the nucleocapsid [16]. The helical curvature of a given linear nucleocapsid is heterogeneous (e.g., the number of subunits per turn is variable) [17]. Unlike the strict helical nucleocapsid, the nucleocapsid is not disrupted even when it is completely relaxed [16]. The nucleocapsid of a negative-strand RNA virus (NSV) possesses such an architecture as can be seen with the nucleocapsid of vesicular stomatitis virus (VSV), a common laboratory NSV (Figure 1) [18,19].

For NSVs, the structure of the capsid subunits within the same virus order can be superimposed with each other, generally speaking [18]. Between the orders, only a similar topology may be recognized in the capsid subunits. Here we will analyze the assembly of linear nucleocapsids by closely examining representatives of the major NSV orders, *Mononegavirales* (VSV), *Bunyavirales* (Bunyamwera virus, BUNV), and *Articulavirales* (Influenza virus, IFV). We will also examine a representative of the positive-strand RNA virus (PSV) genus *Potexvirus* (Potato virus X, PVX), which also forms a linear nucleocapsid. The capsid proteins examined contain structural features of conserved protein folding topology, unique inter-subunit interactions, and efficient encapsidation of the nucleic acid genome. The helical curvature allows the linear nucleocapsid to be compacted more tightly within a virus particle. More importantly, the nucleocapsid of NSVs serves as the template for viral RNA synthesis without releasing the nucleic acid genome from the nucleocapsid. This mechanism of transcription/replication is different from all other known viruses.

## 2. Materials and Methods

Coordinates of all structures were downloaded from RCSB PDB [21]. PDB codes used for VSV were 7UWS, 3PU1, and 5A22. The PDB codes used for BUNV were 3ZLA and 7AOY. The PDB code for IFV was 9BWZ, and for PVX was 6R7G.

Structural drawings for all structures were prepared with PyMol version 1.3 (Schrödinger, LLC, New York, NY, USA) [22].

## 3. Results

### 3.1. Subunit Structure

**Figure 2:**
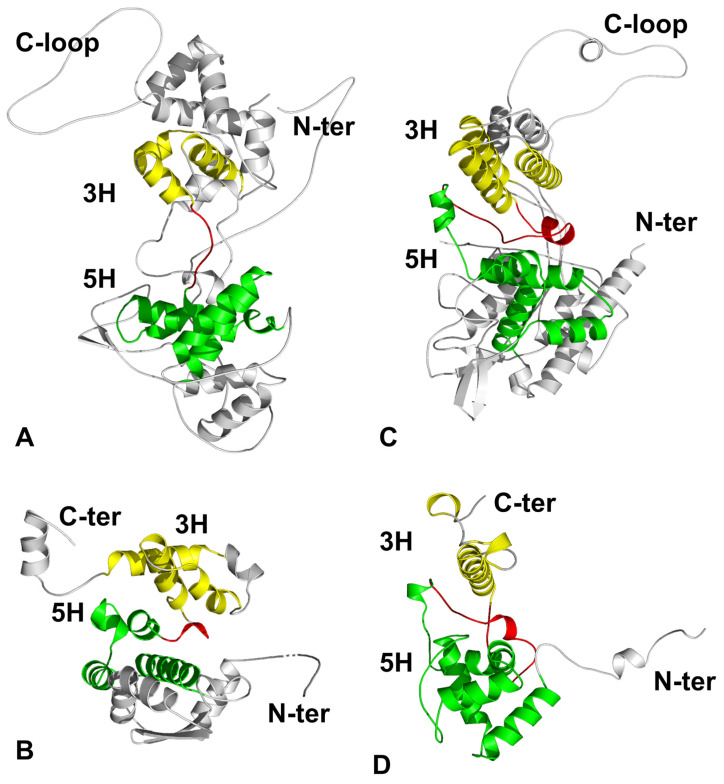
Structure of capsid subunits from four different viruses. (**A**) VSV (PDB 3PU1 [26]). (**B**) BUNV (PDB 3ZLA [27]). (**C**) IFV (PDB 9BWZ [28]). (**D**) PVX (PDB 6R7G [29]). Across all panels, the 5H motif in the NTD is colored green, and the 3H motif in the CTD is colored yellow. The linker connecting the two helix bundles is shown in red. The N-terminus and the C-loop are also indicated for each structure.

In icosahedral viruses, the main capsid protein subunits share a common fold known as a β-barrel (also known as a double jelly roll) [23,24,25]. Similarly, the major protein subunit in the linear viral nucleocapsids also contains a conserved topological fold that we termed the 5H + 3H fold (Figure 2) [18]. The capsid protein structure of viruses in Phylum *Negarnaviricota* shares high structural homology among members in the same order, and retains less homologous but still recognizable structural similarities between orders [18]. The capsid subunit is composed of two domains, the N-terminal domain (NTD) and the C-terminal domain (CTD). There are five helices (5H) arranged with a conserved topology in NTD, whereas there are three helices (3H) arranged in a conserved topology in CTD. The 5H and 3H motifs are connected by a single polypeptide linker. In this context, topology is defined by the conserved sequential connectivity of the secondary structural elements, regardless of the homology of their three-dimensional structures.

**Figure 3:**
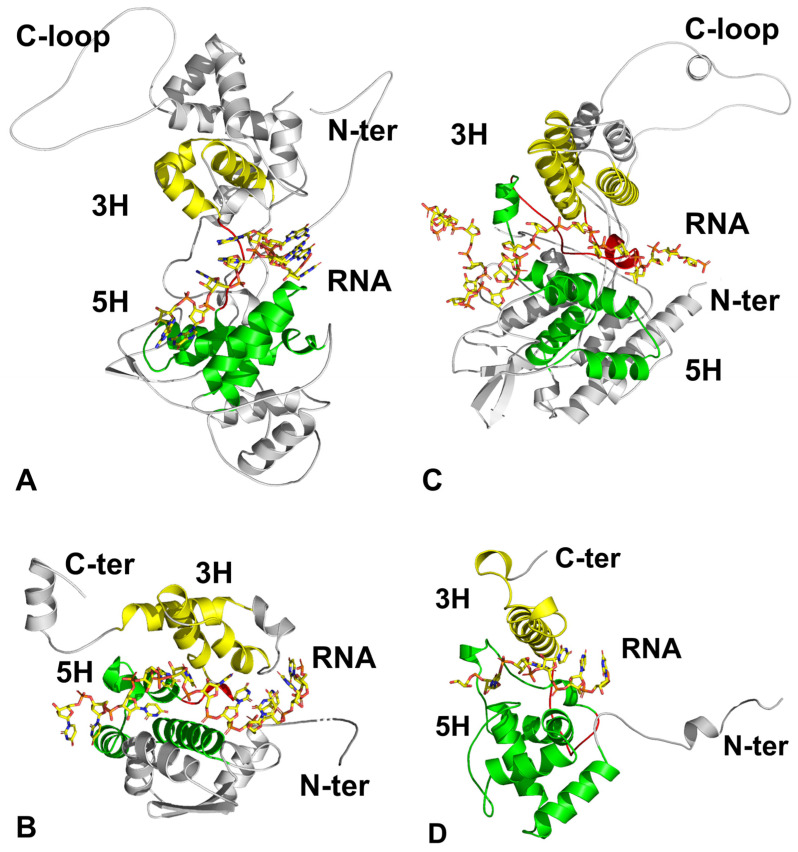
RNA sequestered by capsid subunits. The structures (**A**–**D**) illustrate the capsid subunit from four different viruses with associated sequestered RNA. RNA is shown as sticks, with carbon colored yellow, oxygen colored red, nitrogen colored blue, and phosphorus colored orange. (**A**) VSV (PDB 3PU1 [26]). Nine nucleotides (G) are shown. (**B**) BUNV (PDB 3ZLA [27]). Eleven nucleotides (U) are shown. (**C**) IFV (PDB 9BWZ [28]). The RNA backbone for 19 nucleotides is shown. (**D**) PVX (PDB 6R7G [29]). Six nucleotides (U) are shown. In all representations, the 5H motif in NTD is colored green and 3H motif in CTD is colored yellow. The linker between the two motifs is colored red. The N-terminus and the C-terminus of the capsid subunit are also labeled for reference.

In NSVs, the nucleic acid genome is fully encapsidated in the nucleocapsid, and there is a fixed number of nucleotides associated with each capsid subunit. In VSV, nine nucleotides are associated with each capsid subunit (Figure 3A). The bases of these nucleotides are completely sequestered between the two domains of the capsid subunit. Specifically, three bases are stacked and face the interior of the subunit, while five others are stacked and face the opposite direction, with the ninth nucleotide standing unstacked. In the capsid subunit of BUNV, there are 11 nucleotides associated with each capsid subunit (Figure 3B) [27,30]. Ten of these nucleotides are sequestered by the capsid subunit, with three bases facing the interior and seven bases facing the exterior. Interestingly, one nucleotide, along with the next connecting ribosylphosphate group, spans the gap between the two neighboring subunits. This structural feature exposes part of the RNA genome and renders the capsid susceptible to disassembly when the RNA genome is excised by RNase [31]. Conversely, the nucleocapsid of those viruses in which all bases are sequestered produces an empty capsid when the RNA genome is removed by RNase treatment [32,33]. In the capsid subunit of IFV, there are about 20 nucleotides associated with each capsid subunit (Figure 3C) [28,34]. Two or more nucleotides may be exposed between two neighboring subunits. RNase treatment of the IFV nucleocapsid also results in degradation of the nucleocapsid [35].

Interestingly, despite a lack of sequence homology and apparent functional relationship with NSVs, the capsid subunit fold in the PSV genus Potexvirus shares structural homology with that of NSV and also contains the 5H + 3H fold (Figure 2D) [18,29,36,37,38]. This indicates that this fold shares the evolutionary origin for the linear nucleocapsid to encapsidate a single chain of the RNA genome. Potexvirus (e.g., PVX or PVY) contains five nucleotides that are associated with each capsid subunit fully sequestered (Figure 3D) [29,38]. Similar to VSV, RNase treatment of PVX resulted in an empty capsid [39]. The nucleocapsid of PVX is the intact virus particle, with no other viral components. The structural properties of PVX capsid subunits are inherently suitable for encapsidating the genomic RNA into the nucleocapsids, like in other NSVs.

### 3.2. Architecture of the Linear Nucleocapsid

The linear nucleocapsid is built through intricate interactions between the capsid subunits and the nucleic acid genome. In the nucleocapsid of VSV, there are extensive interactions between the capsid subunits and between the encapsidated RNA strand and the capsid subunits (Figure 1). First of all, there are close lateral side-by-side interactions between the subunits, especially between the neighboring CTDs. Disruption of the side-by-side interactions between CTDs abolished the assembly of the nucleocapsid [32]. Furthermore, the extended N-terminus and a large loop (C-loop) in CTD also have multiple interactions among the four consecutive adjacent subunits. The C-loop in subunit (n) engages the back of the CTD in subunit (n + 1), whereas the N-terminus in subunit (n) attaches the back of the CTD in subunit (n − 1). In addition, the N-terminus in subunit (n) interlocks with the C-loop in subunit (n − 2). The interactions among the capsid subunits make it possible to assemble an empty capsid without the RNA strand being encapsidated [32]. Upon encapsidation of the RNA strand, there are interactions between bases of the nucleotides and residues of the capsid subunit, as well as between the backbone phosphate groups and the charged residues of the capsid subunits [40]. The interactions between the RNA strand and the capsid subunits further stabilize the linear nucleocapsid, even though the encapsidated RNA strand is not required for the capsid assembly [26,33]. The nucleocapsid of members in the order *Mononegavirales* all feature extensive interactions among the capsid subunits and the nucleic acid genome, which results in a relatively high stability that tightly protects the encapsidated RNA [32,41,42,43,44,45,46,47].

**Figure 4:**
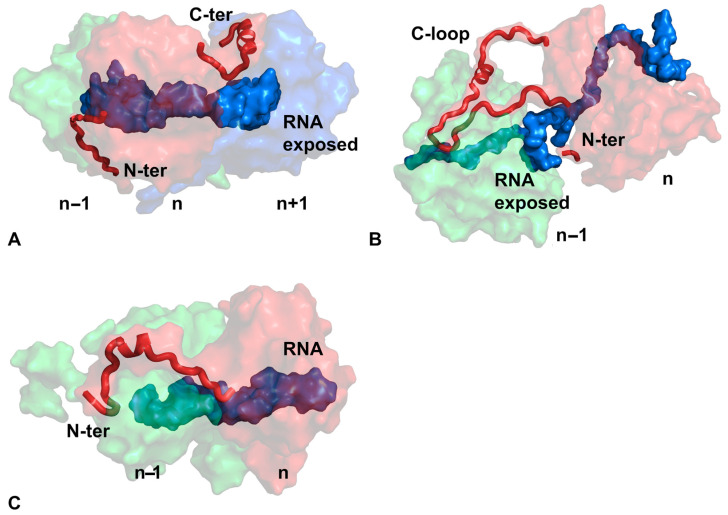
Architecture of the linear nucleocapsid. (**A**) Nucleocapsid of BUNV (PDB 3ZLA [27]). Three capsid subunits are included. (**B**) Nucleocapsid of IFV (PDB 9BWZ [28]). Two capsid subunits are included. (**C**) Nucleocapsid of PVX (PDB 6R7G [29]). Two capsid subunits are included and no RNA is exposed. In all panels, RNA is colored blue, and exposed RNA is labeled. Structural extensions involved in inter-subunit interaction (N-terminus, C-terminus, or C-loop) are highlighted in red. Subunit positions are labeled at the bottom. For VSV, see Figure 1.

In the nucleocapsid of BUNV, the N-terminus of the capsid subunit (n) interacts with the NTD of subunit (n − 1), whereas the C-terminus of subunit (n) interacts with the CTD of subunit (n + 1) (Figure 4A). However, the neighboring subunits lack direct lateral side-by-side contacts. This gap exposes part of the RNA genome, and the encapsidated RNA strand is essential for maintaining the integrity of the linear nucleocapsid [31]. Likewise, the C-loop (or tail loop) in the IFV capsid subunit (n) interacts with subunit (n − 1), and the N-terminus (disordered in the reported structure) could be predicted to possibly interact with subunit (n − 1) based on the orientation of the last ordered N-terminal residue of subunit (n) (Figure 4B). There are no side-by-side interactions between the capsid subunits in the IFV nucleocapsid, consistent with its sensitivity to RNase treatment [35,48].

In the PVX nucleocapsid, the N-terminus of subunit (n) interacts with the NTD of subunit (n − 1) (Figure 4C). In contrast to IFV and BUNV, the lateral side-by-side interactions between subunits in the PVX nucleocapsid are extensive, and there is no extended C-loop or C-terminus in its capsid subunit. The extensive interactions between the capsid subunits account for the stability of the empty capsid when its encapsidated RNA genome is removed by RNase [39,49].

### 3.3. Helical Curvature of the Linear Nucleocapsid

In many reports, NSV nucleocapsids are described as helical; however, a strict helical symmetry requires both rotational symmetry combined with translation along a central axis for assembly of the helical nucleocapsid. However, this strict helical geometry is not a prerequisite for the assembly of the linear nucleocapsid. These nucleocapsid structures merely resemble a helix superficially, rather than adhering to the mathematical helical symmetry [7,50,51,52,53]. In the actual structure, each subunit in the nucleocapsid may adopt a slightly different orientation and does not align rotationally. Therefore, one subunit may not be perfectly superimposed onto another through a conventional helical symmetry operation [20,28,31]. Furthermore, the NSV nucleocapsid often loses its appearance of a helical rod upon release from the virus particle [16,31]. A strict helical symmetry requires precise helical geometry that is functionally unalterable for nucleocapsid assembly, a criterion that the linear nucleocapsid fails to meet.

**Figure 5:**
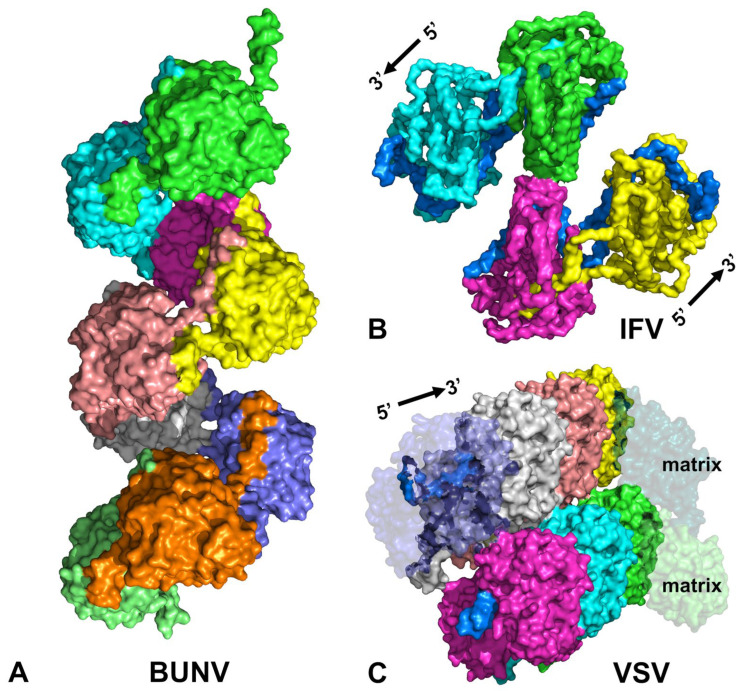
Helical curvature of nucleocapsids. (**A**) Nucleocapsid of BUNV (PDB 7AOY [31]). Nine capsid subunits are shown without encapsidated RNA. A wide groove indicates that there is no interaction of subunits between helical turns. (**B**) Nucleocapsid of IFV (PDB 9BWZ [28]). Four capsid subunits are arranged in a double helical geometry. The encapsidated RNA is colored blue. RNA in the top two subunits runs from 5′ end to 3′ downwards, whereas RNA in the bottom two subunits runs from 5′ end to 3′ upwards. The green subunit in the top strand contacts the magenta subunit in the bottom strand. (**C**) Nucleocapsid of VSV (PDB 7UWS [20]). Two turns of the nucleocapsid are shown, with four capsid subunits in the top turn and three capsid subunits in the bottom turn. The subunits in the bottom turn are staggered (interdigitally shifted) by 0.5 units relative to the subunits in the top turn. Two subunits of the associated matrix protein are also depicted. The helical curvature and the gap between the two turns of the VSV nucleocapsid are maintained by the interactions between the matrix subunits. The encapsidated RNA is shown in blue. In all panels, individual capsid subunits are colored differently to facilitate the visualization of their spatial arrangement and staggering.

In the BUNV nucleocapsid, the capsid subunits are arranged around a wavy helical axis (Figure 5A). The helical segments are short, following a zig-zag trajectory [31]. The helical curvature of different segments of the BUNV nucleocapsid is variable. The structural model for the BUNV nucleocapsid was constructed by fitting the crystal subunit structure into low-resolution cryoEM 3D density maps (Figure 5A) [31]. The terminal regions of capsid subunits involved in interactions between subunits were modeled with flexible conformation. Due to the absence of strict helical symmetry, no RNA strand could be built in this low-resolution model. The helical curvature is not required for the assembly of BUNV nucleocapsid but rather confers flexibility and compatibility.

The absence of strict helical symmetry confers a distinct advantage that allows the nucleocapsid to be compacted when packaged in the virus particle. The degree of helical curvature is imposed by the interactions between the subunits, which may be modulated by the environmental conditions, such as ionic strength. The helical axis may be bent in order to accommodate the nucleocapsid in the virus particle. In IFV, the nucleocapsid is shaped as a double-stranded, flexible helical rod, involving interactions between the subunits in the opposite strands (Figure 5B) [28]. With this geometry, the nucleocapsids are more compact, suited for packaging in the virus particle.

In the VSV virion, the capsid subunits are arranged in a unique fashion. The favorable helical curvature for the VSV nucleocapsid is to have about 10 subunits in one turn [19,20]. However, the subsequent turn must wrap around the first turn, which requires about 18 (17.5) subunits to span the increased diameter. During this transition, the orientation of the subunits becomes more horizontally tilted, compared to the previous turn. This progression continues until the subunits between consecutive turns are parallel to each other in the trunk region. It takes nine turns to transform from the tip to the trunk in the virion. This arrangement results in a bullet shape of the VSV nucleocapsid. Notably, the nucleocapsid alone forms a bullet shape when favorable subunit interactions are possible at pH 5 in a low salt buffer [16]. In the VSV particle, however, the bullet geometry is constructed by interactions of the capsid subunits with the subunits of the matrix protein (Figure 5) [20], as there is no interaction between turns of the capsid subunits.

### 3.4. Nucleocapsid as the Template for Viral RNA Synthesis

A defining characteristic of viral RNA synthesis of NSV is that the template is not naked genomic RNA, but a protein–RNA complex of the nucleocapsid [54,55,56,57]. The viral RNA-dependent RNA polymerase (vRdRp) initiates and elongates viral RNA synthesis while the genomic RNA remains encapsidated in the nucleocapsid. After vRdRp completes the viral transcription or replication, the assembly of the nucleocapsid is restored.

Since the bases of the genomic RNA are sequestered within a nucleocapsid, the RNA genome must be dissociated, at least locally, from the capsid subunits to serve as a template for viral RNA synthesis. The enzymatic activities required for viral RNA synthesis are distributed in different domains of vRdRp [58,59,60,61,62,63,64,65,66,67]. Current observations of the template RNA in the NSV vRdRp structure have involved only an isolated RNA, not a protein–RNA complex [68,69,70,71,72,73,74,75,76]. In structures of the vRdRp L protein, the domains harboring enzymatic activities for mRNA cap generation, including methyltransferase (MTase), polyribonucleotidyltransferase (PRNTase), cap binding domain, or cap-dependent endonuclease, are disordered in many cases, indicating a flexibility that allows reorientation of these domains. The structure of VSV vRdRp in complex with the nucleocapsid has been determined, showing vRdRp peripherally attached to, but not engaged with, the nucleocapsid [28,77,78]. Such peripheral attachment of vRdRp to the nucleocapsid is unprohibited when the nucleocapsid retains a helical curvature, without the release of the genomic RNA. On the other hand, the structural geometry of the nucleocapsid and vRdRp suggests that for vRdRp to gain access to the genomic RNA in the nucleocapsid, the helical curvature of the nucleocapsid must be relaxed. Indeed, it has been observed that the cofactor of vRdRp binds the mumps virus nucleocapsid to induce relaxation of its helical curvature [33].

Structural analyses of the NSV vRdRp in complex with the template RNA reveal that the RNA is positioned in the canonical fingers–palm–thumb motif typical of polymerases [55,72,73,79]. For instance, the ten-nucleotide RNA segment is visualized in the L-VP35 complex of the Ebola virus polymerase [70]. VP35 serves as a polymerase cofactor that attaches the polymerase (L) to the nucleocapsid template [80]. Although the PRNTase domain was ordered in the L structure, the rest of the C-terminal region in L, including the MTase, was not observed in this structure, but was observed in a homologous L structure of VSV [59]. The viral PRNTase and MTase are responsible for adding the cap to viral mRNAs. If the viral genomic RNA is simply threaded through the active site of vRdRp, no structural change is required in vRdRp. However, the promoter for transcription may not be located at the 3′ end of the RNA genome. It has been shown that the VSV vRdRp initiates transcription at an internal genomic promoter [81]. This necessitates that vRdRp must recognize the transcription promoter by accessing the internal sites of genomic RNA sequestered in the nucleocapsid, which implies that vRdRp must induce opening in the nucleocapsid to unveil the sequestered RNA. Furthermore, a coordinated mechanism is required to release the template RNA from the nucleocapsid during elongation and to translocate the polymerase along the nucleocapsid while retaining the RNA genome in the active site. It has been shown that interactions between the gating helix of the nucleocapsid protein (N) that sequesters the RNA genome in nucleocapsid and the L protein of VSV are required for viral RNA synthesis [82]. This functional requirement is supported by the homologous helix in the mumps virus nucleocapsid protein, which becomes disordered when the genomic RNA is removed [83]. Mutations in this homologous helix also compromised viral RNA synthesis by the L protein, as observed in VSV N protein [82,83]. In the case of the L protein from La Crosse virus, large rotational conformational changes in the endonuclease domain and the C-terminal region (containing the cap binding domain) were associated with switching from a template-free state to transcription initiation and then elongation in order to open the channels in the L protein for transcription activities [72].

**Figure 6:**
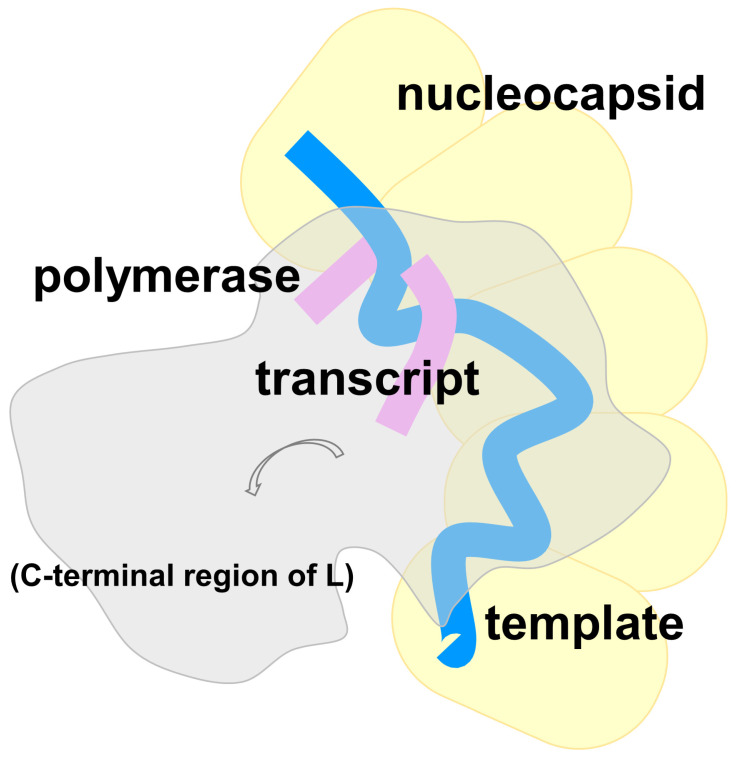
Hypothetical drawing of the viral RNA synthesis bubble. The VSV polymerase L is rendered as a white area. The C-terminal region of the L protein containing PRNTase and MTase was hypothetically rotated to the left (arrow). The nucleocapsid subunits, represented by five yellow boxes, is depicted. The RNA template is colored blue, and the RNA product (e.g., transcript) is colored pink in the hypothetical reaction intermediate.

Based on data from various structural and functional studies, it is postulated that the polymerase L must directly interact with the nucleocapsid protein to induce direct exposure of the genomic RNA into the active site. The nucleocapsid protein and the polymerase L forms a bubble, a transient structure in which the viral RNA synthesis may take place. In VSV L, for example, a hypothetical rotation of the C-terminal region of L starting at the PRNTase domain could create the space for bubble formation (Figure 6). Concurrently, the helical curvature of the nucleocapsid should be relaxed for the polymerase L to gain access to the sequestered genomic RNA.

In PVX, the genomic RNA is positive sense, meaning it serves directly as the RNA used for protein translation. Following host cell entry, the genomic RNA is completely dissociated from the virus particle. The protein-free RNA is then utilized for protein translation and RNA replication [39].

## 4. Discussion

We have shown that the genomic RNA in NSV nucleocapsids is sequestered by a distinct, conserved 5H + 3H fold in the capsid subunit. Variations in the orientation of the 5H and 3H motifs directly alter the shape and the size of the RNA cleft among NSVs [40]. In the case of VSV, the 5H and 3H motifs are more open, resulting in a broad pocket, while BUNV and IFV have a narrower, shallower cleft (Figure 3). Viruses in the order *Bunyavirlales*, in particular, have a very narrow cleft, which may aid in packaging the segmented genome by influencing the nucleocapsid morphology [79].

In the nucleocapsid, the capsid subunits are held together with extensive intermolecular interactions. The capsid subunit (n) in VSV links together three neighboring subunits, (n + 1), (n − 1), and (n − 2), along the linear nucleocapsid. However, NSV with segmented genomes, such as IAV and BUNV, have fewer molecular interactions between neighboring subunits. The NSV nucleocapsid may appear to be helical, but it lacks strict cylindrical symmetry, with capsid units that do not align rotationally. In fact, the symmetry involved in the assembly of the NSV capsid subunits is translational, where the neighboring capsid subunits are moved in a specific direction without rotation and still appear aligned with the starting subunit. The curvature of these nucleocapsids is heterogeneous, and it changes when the surrounding conditions are changed. Through its variable curvature and conserved fold, the linear capsid provides a novel scheme to protect the genome and regulate exposure of RNA during viral RNA synthesis. While it has been shown that the stability of the nucleocapsid influences the rate of viral synthesis, it is still unclear what role the flexibility of the nucleocapsid plays during infection [84,85].

The unique mechanism of NSV transcription and replication lends itself well to laboratory manipulation. Since the RNA–protein complex of a nucleocapsid is linear, regions in the RNA genome can be removed or added [86]. Unlike with icosahedral symmetry, the one-dimensionality of the translational symmetry allows the genomic RNA to be encapsidated and protected at any length. The linear nucleocapsid serves as the template for viral RNA synthesis, thus keeping RNA sequestered from cellular proteins. In another study, researchers were able to generate recombinant VSV encoding foreign glycoproteins by providing the required proteins of the replication complex in a plasmid-based system [87]. These platforms could provide insight into expression and replication by more virulent viruses and aid in early antiviral screening.

## Data Availability

The raw data supporting the conclusions of this article will be made available by the authors on request.

## Abbreviations

The following abbreviations are used in this manuscript:

3H	three helices
5H	five helices
BUNV	Bunyamera virus
CTD	C-terminal domain
C-ter	C-terminus
IFV	Influenza virus
L	polymerase
MTase	methyltransferase
N	nucleocapsid protein
NSV	Negative-strand RNA virus
NTD	N-terminal domain
N-ter	N-terminus
PDB	Protein Data Bank
PRN-Tase	GDP polyribonucleotidyltransferase
PSV	positive-strand RNA virus
PVX	Potato virus X
PVY	Potato virus Y
RNA	ribonucleic acid
vRdPp	viral RNA-dependent RNA polymerase
VSV	vesicular stomatitis virus
